# Influence of Occupational and Environmental Exposure to Low Concentrations of Polychlorobiphenyls and a Smoking Habit on the Urinary Excretion of Corticosteroid Hormones

**DOI:** 10.3390/ijerph13040360

**Published:** 2016-03-25

**Authors:** Maria Nicolà D’Errico, Piero Lovreglio, Ignazio Drago, Pietro Apostoli, Leonardo Soleo

**Affiliations:** 1 Section of Occupational Medicine “Enrico Carlo Vigliani”, Interdisciplinary Department of Medicine, University of Bari, Piazza G. Cesare 11, Bari 70124, Italy; marianicola.derrico@uniba.it (M.N.D.); ignazio.drago@gmail.com (I.D.); leonardo.soleo@uniba.it (L.S.); 2 Section of Occupational Medicine and Industrial Hygiene, Department of Experimental and Applied Medicine, University of Brescia, Piazzale Spedali Civili 1, Brescia 25123, Italy; pietro.apostoli@unibs.it

**Keywords:** polychlorobiphenyls, corticosteroid hormones, tobacco smoke, glucuronidation, endocrine disruptor

## Abstract

The effects of occupational exposure to low concentrations of polychlorobiphenyls (PCBs) on the urinary excretion of corticosteroid hormones were evaluated, taking into account the influence of cigarette smoking. The study included 26 males working as electrical maintenance staff in a steel factory, previously exposed to a mixture of PCBs (exposed workers), and 30 male workers with no occupational exposure to PCBs (controls). Serum PCBs (33 congeners), urinary 17-hydroxycorticosteroids, 17-ketosteroids (KS) and pregnanes, and their respective glucuronidated and sulfonated compounds, were determined for each subject. PCBs were significantly higher in the exposed workers than controls, and were correlated with age. Both the urinary concentrations of the total 17-KS and pregnanes, and those of some single steroids and their glucuronidated compounds, were significantly lower in the exposed workers than controls, but higher in smokers than the non-smokers + ex-smokers. Two-way analysis of variance showed a negative association between serum PCBs and both total glucuronidated 17-KS and total and glucuronidated pregnanes, and a positive association between cigarette smoking and both total and glucuronidated 17-KS. PCBs seem to act as endocrine disruptors by reducing the urinary excretion of corticosteroid hormones, particularly of the glucuronidated fraction. Cigarette smoking could boost these effects of PCBs in smokers.

## 1. Introduction

Polychlorobiphenyls (PCBs) are chlorinated compounds consisting of 209 possible congeners, depending on the position and number of chlorine atoms on the biphenyl. Owing to their unique properties of heat resistance and chemical stability, PCBs were ubiquitously used in many industrial and commercial applications as insulating fluids in electric transformers and other devices, and as additives in pesticides, flame-retardants, plastic materials and paints. Later, they were discovered to pose a public health hazard due to their toxic effects in humans and in animals, depending on the PCB congener, as well as their biomagnification in the food chain and persistence in the environment. For this reason, their production and use has been banned or severely restricted in most countries since the 1970s. Nowadays, therefore, occupational exposure to PCBs is probably limited to certain situations, such as PCBs waste disposal plants, or maintenance of contaminating appliances still in use, or else laboratories devoted to analytical determinations of the presence of these congeners [1,2,3,4]. Moreover, even though PCBs have been banned, non-occupational exposure to PCBs may still occur in the general population due to their widespread distribution in the environment and food chain.

Individual PCBs can act on different receptors including aryl hydrocarbon receptor (AhR), whose activation is one of the key events linked to carcinogenesis mediated by dioxin-like PCBs, as well as the nuclear human steroid and xenobiotic receptors (SXR), and possibly has an endocrine disruption action on the reproductive system. Previous studies have shown that PCBs can variably influence the levels of corticosteroid hormones. In fact, in some studies on experimental animals administered mixtures of PCBs by different routes, an increase in serum levels of corticosterone was reported, in others a reduction in the serum levels of corticosterone, dehydroepiandrosterone (DHEA) and DHEA sulfate (DHEA-S), while in yet other studies no effect was observed [4]. In humans, too, environmental exposure to PCBs has revealed different effects, ranging from a positive association with serum DHEA-S in children, to a reduction of the urinary metabolites of 17-ketosteroids (KS) and 17-hydroxycorticosteroids (17-OHCS), of sulfonated compounds for 17-OHCS and of the sulfonation percentage of 17-KS and 17-OHCS [5,6,7].

However, many other factors, either endogenous or related to lifestyle, can affect the metabolism of the corticosteroid hormones [8,9]. Among these factors, cigarette smoking has been shown to increase the plasma levels of cortisol and adrenal androgens such as androstenedione and DHEA-S, mediated by the pituitary hormones [10].

From the reports in literature, therefore, it appears that studies of the relation between the urinary excretion of corticosteroid hormones and exposure to PCBs or cigarette smoking, respectively, have elicited contrasting findings. For this reason, the aim of the present study was to investigate the influence of PCBs and a smoking habit on the urinary excretion of the metabolites of the corticosteroid hormones in subjects with occupational exposure to low concentrations of these toxicants.

## 2. Materials and Methods

### 2.1. Study Subjects

All 26 male workers still in active service at a steel factory in Taranto (Italy), who worked as electrical maintenance staff and in the past had carried out maintenance of transformers in which a mixture of PCBs (brand name “Apirolio”, whose precise composition in terms of PCB congeners is not known) was used as dielectric oil, were enrolled in October to November 2009 as subjects with occupational exposure to PCBs (Exposed workers). They had carried out transformers maintenance activities during the period from 1980 to 2005. They were compared with 30 male workers at a different company in Bari, about 90 km from Taranto, that produces metal section bars, with no current or previous occupational exposure to PCBs (Controls), enrolled in the study for the same period.

All participants were administered, by trained medical staff in the infirmaries of the two factories, a questionnaire probing lifestyle, smoking habit, alcohol consumption, diet, hobbies that might expose to PCBs, and medical history to exclude endocrine dysfunction. All workers had been informed of the study aims and gave prior written informed consent to take part. The study was conducted in accordance with the Declaration of Helsinki.

### 2.2. Blood and Urine Sampling

On the day after filling out the questionnaire, between 7:00 a.m. and 10:00 a.m., a blood sample was taken from all the fasting exposed workers and controls, using Vacutainer SST II Advance test tubes. After blood centrifugation, the serum was distributed in two test tubes, to determine PCBs, and total cholesterol and triglycerides used to adjust the PCBs concentration levels, respectively. Immediately after blood sampling, all subjects supplied a urine sample in sterile containers for urinary steroids determinations. The sera used to determine PCBs and the urine samples were immediately frozen, preserved at −20 °C and sent to the toxicology lab; the analyses were made within 10 days of sample collection. The sera for determining total cholesterol and triglycerides were analyzed immediately after collection. 

### 2.3. Analytical Methods

*PCBs and lipids*: The serum of all the subjects studied was examined for 33 PCB congeners (28, 31, 52, 74, 77, 81, 99, 101, 105, 114, 118, 123, 126, 128, 138, 146, 153, 156, 157, 167, 169, 170, 172, 177, 180, 183, 187, 189, 194, 196 + 203, 201, 206, and 209) [4]. The analytical determination of PCBs was performed at the Laboratory of Occupational Hygiene and Toxicology of the Department of Experimental and Applied Medicine of the University of Brescia (Italy) using a Hewlett-Packard 6890N gas-chromatograph coupled with an MSD HP 5973. For the analytical procedure, the modified version of the PCB measurement method described by Turci *et al.* [11] was adopted. Assessment of the intra-day precision of the method, based on five pooled human sera, evinced a coefficient of variation ranging from 3.0% to 6.6% [12]. PCB congener 30 was used for internal standard. The limit of quantification (LOQ) for each congener was equal to 0.1 ng/mL. The serum PCBs, measured as ng/mL, were adjusted for the total serum lipids (TSL) and expressed as ng/g lipids, applying the formula: TSL (g/L) = 2.27 × total cholesterol + triglycerides + 0.623 [13]. The total PCBs in the exposed workers and controls were obtained by summing the results exceeding the LOQ for each congener. The TEQ content has not been calculated because the majority of dioxin-like congeners were below the LOQ and the only dioxin-like congener present in concentrations exceeding 5% of the total was the congener 118 [14].

Total cholesterol and triglycerides were determined at the Occupational Medicine Section “E. C. Vigliani” of the Interdisciplinary Department of Medicine of Bari University, Italy, using the Clinical Chemistry System ILab 300 Plus at the Instrumentation Laboratory.

*Urinary steroids*: Urinary steroids were measured by gas chromatography at the Laboratory of Occupational Hygiene and Toxicology of the Department of Experimental and Applied Medicine of the University of Brescia, Italy [15]. The identification of GC peaks was performed using mixtures of standard hormones obtained from Sigma Chemical Co., St Louis, MO, USA. Total urinary steroids and sulfonated portion were determined by comparing hormone concentrations obtained using enzymes capable of hydrolyzing both the glucuronidated and sulfonated compounds (S.H.P.-helix pomatia juice from the Industrie Biologique Francaise, Gennevilliers, France) with those obtained from bovine liver *β*-glucuronidase (Sigma Chemical Co., St Louis, MO, USA), an enzyme that has only a glucuronidasic activity [16]. The following urinary metabolites of steroids were determined: 17-OHCS: 11-deoxycortisol (THS), 11-dehydroxicortisone (THE), 11-hydroxycortisol (THF), α-11-hydroxycortisol (α-THF), 11-dehydrocorticosterone (THA), corticosterone (THB), α-corticosterone (α-THB), cortolone, β-cortolone, cortol, β-cortol; 17-KS: androsterone, DHEA, etiocholanolone, 11-hydroxyandrosterone, 11-ketoandrosterone, 11-ketoetiocholanolone; Pregnanes: pregnanediol, α-pregnanediol, d5-pregnanediol, α-d5-pregnanediol, pregnanetriol, d5-pregnanetriol, and pregnanetriolone.

The limit of detection of 17-OHCS was 0.02 mg/L, and of 17-KS and Pregnanes 0.01 mg/L; coefficients of variations were 12.6% for 17-OHCS, 8.3% for 17-KS and 5.1% for Pregnanes. The urinary concentrations are reported as total steroids, sulfonated and glucuronidated steroids. All the urine samples showed urinary creatinine values ranging between 0.3 g/L and 3.0 g/L, within the WHO recommended limits for biological samples to be judged acceptable [17].

### 2.4. Statistical Analyses

Statistical analyses were made using the SPSS program (version 14.0, Chicago, IL, USA). A normal distribution of all variables was verified using the Kolmogorov–Smirnov test. Not normally distributed variables were analyzed by parametric tests after logarithmic transformation or non-parametric Mann–Whitney test. Analyses of correlations were made with the Spearman test. The level of significance was set at *p* < 0.05.

## 3. Results

There were no differences in general characteristics, lifestyle and consumption of foods with a particularly high content of PCBs between the exposed subjects and controls (Table 1).

Table 2 shows the serum concentrations of total PCBs, of congeners 118, 138, 153, 170, 180 and 187, that were present in concentrations exceeding 5% of the total, of the sum of these six congeners, as well as the percentage of each congener and of the sum of the six congeners with respect to the total PCBs, in the two groups. The concentration of total PCBs was obtained in exposed subjects *versus* controls, being the sum of 25 *versus* 11 of the 33 detectable congeners. The concentrations of total PCBs and of the various congeners and the sums of the six congeners were significantly higher in exposed subjects than controls, while the percentage sum was significantly higher in the controls. Similar findings were obtained when the results were expressed as units of nanograms per milliliter (data not shown); therefore, in the subsequent analyses only serum PCBs expressed as units of nanograms per gram of lipids was considered.

A significant positive correlation between the total PCBs and age was shown, both when analyzing the exposed workers (*r* = 0.46, *p* < 0.05) and controls (*r* = 0.75, *p* < 0.001) separately and together (*r* = 0.46, *p* < 0.001). In addition, in the exposed workers total PCBs were positively correlated with the duration of exposure (*r* = 0.39, *p* < 0.05).

In the exposed workers the urinary concentrations of total 17-KS (Table 3) and total pregnanes (Table 4) were significantly lower than in the controls, whereas the total 17-OHCS were not different between the two groups (data not shown), and so 17-OHCS were not considered in the subsequent statistical analyses. The same table also shows the different urinary 17-KS and pregnanes determined, as well as the concentrations of the glucuronidated and sulfonated fractions of both total 17-KS and pregnanes and the single steroids of the two families. All single 17-KS showed significantly lower urinary concentrations in the exposed workers than in the controls. The urinary glucuronide fraction of both total 17-KS and each different 17-KS was significantly lower in the exposed workers than the controls, whereas the sulfonate fraction showed this trend only for 11-ketoandrosterone. Of the different pregnanes, pregnanediol, α-d5-pregnanediol, d5-pregnanediol and pregnanetriol showed significantly lower urinary concentrations in the exposed workers than the controls. The glucuronidated fraction was significantly lower in the exposed workers than the controls, not only for the total pregnanes but also for each pregnane analyzed singly and for pregnanetriolone, while the sulfonated fraction of both total pregnanes and each one analyzed singly did not show any difference between the two groups.

A correlation with the total PCBs was verified for the steroids listed in Table 3 and Table 4, which were significantly lower in the exposed workers than in the controls, analyzed both separately and as a single group. A significant negative correlation only in the exposed workers and controls analyzed together was found for total 17-KS (*r* = −0.28, *p* < 0.05), androsterone (*r* = −0.37, *p* < 0.01) and the glucuronide fraction of androsterone (*r* = −0.30, *p* < 0.05), whereas no correlation was found when the two groups were analyzed separately. 

There was no significant correlation in the exposed workers between the duration of exposure to PCBs and the different urinary 17-KS and pregnanes determined (data not shown).

To assess the effect of a smoking habit on the excretion of urinary steroids, the exposed workers and controls were subdivided into two groups, smokers *versus* non-smokers + ex-smokers. Compared with non-smokers + ex-smokers, smokers had significantly higher urinary concentrations of total 17-KS, androsterone, etiocholanolone, 11-ketoandrosterone, 11-ketoetiocholanolone and their glucuronidated compounds, whereas for DHEA and 11-hydroxyandrosterone only the glucuronidated fraction was significantly higher (Supplementary Table S1). Smokers also showed significantly higher levels of total pregnanes, pregnanediol, α-d5-pregnanediol and d5-pregnanediol and their glucuronidated fractions (Supplementary Table S2). Instead, no difference was found between smokers and non-smokers + ex-smokers for the urinary excretion of total 17-OHCS and the metabolites analyzed singly.

A correlation among 17-KS, pregnanes, those glucuronidated fractions found to be significantly higher in the smokers and total PCBs was also analyzed in the smokers and non-smokers + ex-smokers. In the smoker group (Table 5), no correlation was found between total PCBs and the different urinary steroids. By contrast, in the non-smokers + ex-smokers there was a significant negative correlation between total PCBs and total 17-KS, androsterone, etiocholanolone, total pregnanes, d5-pregnanediol and their glucuronidated fractions, except for etiocholanolone. In view of the opposite action exerted by total PCBs and cigarette smoking on the urinary excretion of 17-KS, pregnanes and their glucuronidated fractions, two-way analysis of variance was applied to assess the role of these two risk factors. Only those urinary steroids and their glucuronidated fractions found to be significantly reduced in the exposed workers compared to the controls were analyzed (Table 3 and Table 4), and those significantly increased in the smokers compared to the non-smokers + ex-smokers. A significant association resulted for the 17-KS between exposure both to PCBs and to cigarette smoking and glucuronidated 17-KS, etiocholanolone and etiocholanolone glucuronide, and between cigarette smoking and total 17-KS, androsterone, 11-ketoetiocholanolone and 11-ketoetiocholanolone glucuronide (Table 6). For the pregnanes, a significant association was found between exposure to PCBs and total pregnanes, pregnanediol and the glucuronidated compound and d5-pregnanediol, and between cigarette smoking and α-d5-pregnanediol, d5-pregnanediol glucuronide, pregnanetriol and pregnanetriol glucuronide (Table 7).

## 4. Discussion

The study shows that exposure to low concentrations of PCBs might be associated with a reduced urinary excretion of 17-KS and pregnanes, both total and the single steroid hormones, and of their glucuronidated fractions, whereas cigarette smoking is associated with an increased urinary excretion. Neither of the risk factors affects the urinary excretion of 17-OHCS.

Exposed worker group showed concentrations of total PCBs higher than controls and a partially different profile in terms of the congeners. In fact, the percentage sum of the six congeners exceeding 5% of the total was 100% as median value in the controls and 96.6 in the exposed worker group. These results, however, suggest a different origin of exposure to PCBs for the two groups, although the differences are not so evident because of the time from the last exposure. Total PCBs are also positively correlated with age, and in the exposed workers group, with the years of exposure to PCBs, in agreement with data in the literature reporting a tendency of these compounds to accumulate in the organism with an increasing duration of exposure [4,18].

The reduced urinary excretion of total 17-KS observed in our study is in agreement with the report by Romeo *et al.* [6] in subjects of the general population resident in urban areas near an industrial plant producing PCBs. These subjects had a mean serum concentration of total PCBs of 61.9 ng/mL calculated on 24 congeners, higher than the 10.04 ng/mL, calculated on 33 congeners, observed in our workers exposed to PCBs. These Authors also found a reduced urinary excretion of total 17-OHCS and their sulfonated compound but no difference in the urinary excretion of the total pregnanes and their sulfonated compounds. They did not study the glucuronidated compounds of any of these three classes of urinary steroids. Our study, therefore, even confirming the possible effect of PCBs as endocrine disruptors of the endogenous steroid hormone homeostasis, seems to show some differences from the findings of Romeo *et al.* Such differences lead us to hypothesize that PCBs target and mode of action could be, at least partially, influenced by the exposure level and/or the pattern of congeners exposure, the latter depending on occupational or environmental exposure.

Glucuronidation and sulfonation are catalyzed by the UDP-glucuronosyltransferase (UGT) enzyme family, in the presence of the cofactor uridine diphosfate-glucuronide (UDPglucuronide), and by the sulfotransferase (SULT) enzyme family in the presence of the cofactor 3′-phosphoadenosine-5′-phosphosulfate (PAPS). Apart from the steroid hormones, these enzymes also have various endogenous and exogenous chemical compounds as substrate, including PCBs. At the hepatic level the latter can undergo phase I and II metabolic reactions, as confirmed both *in vitro* and in experimental animals [19,20,21,22]. In particular, after hydroxylation by the P450 cytochromes, congeners with a lower degree of chlorination can be glucuronidated or sulfonated and so eliminated in bile and, to a lesser extent, in urine. Otherwise, they can remain in the blood, or be newly hydroxylated to quinones. By contrast, congeners with greater chlorine content are less susceptible to metabolic reactions and so persist in biological tissues.

The reduction only of the glucuronidated fraction of 17-KS and pregnanes, observed in our exposed workers, suggests that this could have been determined by the PCBs through enzymatic inhibition during the glucuronidation phase. This inhibition could depend on a reduction of UDP glucuronide, that is therefore subtracted from the glucuronidation reactions of the other compounds, as demonstrated by Chan *et al.* [23] in rat hepatocytes treated *in vitro* with a mixture of PCBs (congeners 28, 77, 105, 126, and 153). However, other mechanisms could underlie the inhibitory effect. Highly chlorinated PCB congeners, for instance, and especially PCB 153, are antagonists of SXR and so can inhibit the genetic induction of the phase I and II metabolism enzymes that are physiologically activated by the steroid hormones [24]. This antagonistic effect exerted by PCBs on the SXR receptor could also negatively modulate the induction of the microsomial enzymes [24,25], as described in the literature for the dioxin-like PCBs, mediated by the activation of the AhR [26,27,28]. Finally, a possible direct effect of PCBs on the hepatic enzymes of the phase II metabolism has been described, inhibiting both the glucuronidation and sulfonation of some steroid and xenobiotic hormones [1,2,21,28].

In our study, no significant reduction of the urinary excretion of the sulfonated steroid hormones was observed in the exposed workers. An inhibition of the SULT, and in particular of the hSULT2A1 isoform, has recently been described in some *in vitro* studies that suggested a possible non competitive inhibition mechanism exerted both by a mixture of OH-PCB, probably depending on the availability of the PAPS cofactor, and of PCB quinones, highly oxidating metabolites of the PCBs [29,30,31,32]. However, Van den Hurk *et al.* [33] demonstrated, at the level of the bowel mucosa of the catfish, that hydroxylated PCBs inhibit the UGT enzymes to a greater extent than the SULT. They pointed out that the SULT are enzymes with a high affinity and low capacity while the UGT have low affinity and high capacity. Further study seems necessary to clarify whether the effects on the sulfonation of the steroid hormones by the PCB metabolites observed *in vitro* are also present in man as a consequence of environmental or occupational exposure to low concentrations of PCBs, and whether these can cause an effect of endocrine disruption.

Our results demonstrated, in smokers, a higher urinary excretion of the total 17-KS and total pregnanes, their glucuronidated fractions and of some of the single steroid hormones, in agreement with previous studies [34,35]. This effect could be due either to the toxic action of nicotine or to increased stress effects on the organism induced by the smoking habit [10]. Moreover, cigarette smoke could enhance the expression of several P450 cytochromes [36]. In our study, a smoking habit seemed to have a confounding effect on the inhibition by PCBs of the urinary excretion of 17-KS and pregnanes and, in particular, of their glucuronidated fractions, as shown by the significant correlation between these steroids and the total PCBs in the non-smoker + ex-smoker group but not in the smoker group. This negative effect of PCBs and positive effect of cigarette smoking on the urinary excretion of the steroids was confirmed by two-way analysis of variance.

This study presents some limits that should be considered, including small sample size and the fact that, for company organization reasons, the urinary levels of the corticosteroid hormones were measured on urine spot samples and not on 24 h urine collection samples. Moreover, these determinations were not associated with evaluation of the serum levels.

## 5. Conclusions

Our results clearly indicate that PCBs could have an effect as endocrine disruptors by reducing the urinary excretion of the steroid hormones through inhibiting their glucuronidation and/or through other effects on their metabolic biotransformation pathways. This could likely result in their persistence in the organism. Cigarette smoking, in turn, by stimulating the production of these hormones, could boost their serum concentrations. Together with the reduced urinary excretion caused by the PCBs, this could foster increased biological effects in the organism. To clarify this interaction, and whether causes clinical effects in exposed workers, it could be useful to determine both the urinary and the serum concentrations of the steroids at the same time, bearing in mind that these glucuronidation mechanisms are shared by many other environmental pollutants.

## Figures and Tables

**Table 1:** General characteristics of the subjects exposed to PCBs and the controls.

Variable	Exposed Subjects				Controls			
	N (%)	Mean ± SD	Median	Range	N (%)	Mean ± SD	Median	Range
Age (years)	26	37.0 ± 6.9	34.0	28.0–55.0	30	38.0 ± 8.6	36.0	24.0–55.0
Body Mass Index (kg/m^2^)	26	26.3 ± 3.3	25.8	20.9–36.9	30	25.7 ± 3.4	26.4	16.2–32.9
Occupational exposure to PCBs (years)	26	10.0 ± 6.5	8.0	2.0–25.0	-			
Smoking habit								
Smoker	6 (23.1)				14 (46.6)			
Non-smoker	9 (34.6)				8 (26.7)			
Ex-smoker (>1 year ago)	11 (42.3)				8 (26.7)			
Smokers (cigarettes/day)								
1–10	5 (19.2)				5 (16.7)			
11–20	1 (3.9)				7 (23.3)			
>20	-				2 (6.6)			
Alcohol consumption								
Teetotal	5 (19.2)				1 (3.3)			
<12 g/day	14 (53.9)				17 (56.7)			
≥12 g/day	7 (26.9)				12 (40.0)			

**Table 2:** Serum concentrations of total PCBs, single congeners and the two sums of congeners expressed as units of nanograms per gram of lipids and as percentage of the total PCB, in exposed subjects and controls.

PCBs		Exposed Subjects (N = 26)				Controls (N = 30)			
		Mean ± SD	Median	Range	N_<LOQ_^a^	Mean ± SD	Median	Range	N_<LOQ_^a^
Total	ng/g lipids **	1623.4 ± 384.6	259.0	79.3–19006.0	-	166.3 ± 114.9	126.8	19.3–528.8	-
Congener 118	ng/g lipids *	51.5 ± 109.5	15.5	6.3–564.1	8	-	8.5	5.0–36.5	23
	% *	3.6 ± 3.7	3.6	0.0–16.7		1.5 ± 2.9	0.0	0.0–8.3	
Congener 138	ng/g lipids **	319.6 ± 733.6	66.1	18.4–3666.4	-	39.5 ± 23.2	33.6	8.6–109.4	2
	%	23.2 ± 3.8	23.8	16.3–29.4		24.2 ± 9.4	23.6	0.0–50.1	
Congener 153	ng/g lipids **	381.0 ± 837.8	78.4	36.7–4136.5	-	59.6 ± 33.2	51.9	17.1–164.1	-
	% **	31.4 ± 7.9	29.8	21.3–50.1		40.7 ± 13.7	36.4	30.0–100	
Congener 170	ng/g lipids *	121.9 ± 287.9	22.7	7.0–1410.2	7	-	9.7	5.8–36.5	17
	%	5.6 ± 3.7	7.2	0.0–9.5		3.5 ± 4.2	0.0	0.0–11.1	
Congener 180	ng/g lipids *	329.3 ± 765.7	58.6	19.8–3744.8	-	42.9 ± 23.4	38.0	8.2–109.4	3
	%	23.4 ± 5.1	22.3	14.7–33.3		25.9 ± 12.7	27.3	0.0–66.7	
Congener 187	ng/g lipids *	105.9 ± 254.4	18.9	7.2–1269.2	7	-	9.4	5.0–36.5	19
	%	5.1 ± 3.4	6.1	0.0–9.5		3.3 ± 4.6	0.0	0.0–13.0	
Sum of the 6 congeners	ng/g lipids **	1309.3 ± 2984.4	255.2	109.1–14,791.1	-	-	150.9	67.7–492.3	-
	% **	92.1 ± 9.4	96.6	76.4–100		99.0 ± 2.5	100	90.1–100	

^a^ In the % calculations determinations lower than the LOQ were inserted in the analysis with value 0; * *p* < 0.01; ** *p* ≤ 0.001.

**Table 3:** Urinary concentrations of total 17-KS and the different steroids and their respective glucuronidated and sulfonated compounds in the exposed subjects and controls.

17-KS (mg/L)	Exposed Subjects (N = 26)			Controls (N = 30)		
	Mean ± SD	Median	Range	Mean ± SD	Median	Range
Total ^c^	6.14 ± 3.44	5.78	1.58–14.68	9.86 ± 4.28	8.92	1.38–21.85
Glucuronides ^b^	3.89 ± 3.29	2.97	0.25–13.81	6.85 ± 3.82	6.16	0.16–17.68
Sulfonates	2.24 ± 1.29	2.06	0.15–4.83	3.01 ± 2.08	2.42	0.18–9.21
Androsterone ^b^	2.51 ± 1.31	2.54	0.66–5.67	3.66 ± 1.84	3.27	0.74–10.30
Glucuronide ^a^	1.59 ± 1.31	1.21	0.08–4.80	2.61 ± 1.69	1.91	0.06–7.71
Sulfonate	0.92 ± 0.52	0.89	0–1.85	1.05 ± 0.86	0.83	0–3.92
Etiocholanolone ^c^	1.62 ± 1.02	1.47	0.41–4.40	2.74 ± 1.26	2.49	0.24–6.67
Glucuronide ^c^	1.33 ± 1.07	1.00	0.13–4.40	2.34 ± 1.17	2.10	0.05–5.81
Sulfonate	0.29 ± 0.22	0.27	0–0.78	0.40 ± 0.47	0.24	0–2.04
DHEA ^b^	0.17 ± 0.36	0.03	0.01–1.63	0.26 ± 0.39	0.09	0.01–1.74
Glucuronide ^b^	0.05 ± 0.09	0.02	<0.01–0.38	0.09 ± 0.09	0.06	0.01–0.43
Sulfonate	0.11 ± 0.28	0.01	0–1.25	0.17 ± 0.31	0.04	0–1.42
11-hydroxyandrosterone ^b^	1.43 ± 0.77	1.45	0.17–3.74	2.33 ± 1.58	2.22	0.28–8.84
Glucuronide ^a^	0.61 ± 0.69	0.40	0.01–3.02	1.13 ± 1.06	0.76	0.01–4.47
Sulfonate	0.83 ± 0.49	0.73	0–1.64	1.20 ± 1.00	0.86	0–5.00
11-ketoandrosterone ^c^	0.06 ± 0.05	0.04	0.01–0.25	0.13 ± 0.15	0.09	0.01–0.76
Glucuronide ^a^	0.04 ± 0.04	0.02	<0.01–0.19	0.07 ± 0.07	0.04	0.01–0.36
Sulfonate ^a^	0.02 ± 0.02	0.01	0–0.07	0.06 ± 0.14	0.03	0–0.75
11-ketoetiocholanolone ^c^	0.37 ± 0.32	0.26	0.05–1.42	0.82 ± 0.50	0.80	0.10–2.73
Glucuronide ^c^	0.27 ± 0.31	0.17	0.01–1.40	0.66 ± 0.50	0.51	0.02–2.73
Sulfonate	0.09 ± 0.12	0.05	0–0.44	0.17 ± 0.25	0.10	0–1.13

17-KS: 17-ketosteroids; DHEA: dehydroepiandrosterone. ^a^ *p* < 0.05; ^b^ *p* < 0.01; ^c^ *p* ≤ 0.001.

**Table 4:** Urinary concentrations of the total pregnanes and different steroids and their respective glucuronidated and sulfonated compounds in the exposed subjects and controls.

PREGNANES (mg/L)	Exposed Subjects (N = 26)			Controls (N = 30)		
	Mean ± SD	Median	Range	Mean ± SD	Median	Range
Total ^b^	1.66 ± 1.26	1.37	0.37–5.64	2.58 ± 1.14	2.43	0.22–4.75
Glucuronides ^a^	1.09 ± 1.14	0.68	0.09–5.26	1.87 ± 1.06	1.68	0.09–4.35
Sulfonates	0.57 ± 0.31	0.48	0.15–1.24	0.72 ± 0.47	0.61	0.02–2.19
Pregnandiol ^b^	0.25 ± 0.27	0.20	0.05–1.34	0.37 ± 0.22	0.32	0.04–0.88
Glucuronide ^b^	0.20 ± 0.26	0.12	0.02–1.28	0.31 ± 0.21	0.23	0.02–0.78
Sulfonate	0.05 ± 0.04	0.04	0–0.16	0.06 ± 0.05	0.05	0–0.20
α-pregnanediol	0.08 ± 0.06	0.06	0.01–0.21	0.15 ± 0.17	0.10	0.01–0.61
Glucuronide	0.06 ± 0.05	0.04	0.01–0.20	0.10 ± 0.07	0.08	0.01–0.26
Sulfonate	0.02 ± 0.02	0.01	0–0.06	0.05 ± 0.11	0.01	0–0.39
α -d5-pregnanediol ^c^	0.27 ± 0.32	0.16	0.01–1.56	0.56 ± 0.40	0.50	0.04–1.90
Glucuronate ^c^	0.22 ± 0.28	0.09	0.01–1.29	0.46 ± 0.37	0.43	0.02–1.90
Sulfonate	0.05 ± 0.06	0.04	0–0.27	0.10 ± 0.16	0.05	0–0.77
d5-pregnanediol ^a^	0.10 ± 0.08	0.08	0.01–0.29	0.15 ± 0.08	0.14	0.01–0.33
Glucuronide ^b^	0.04 ± 0.04	0.03	<0.01–0.15	0.08 ± 0.05	0.08	0.01–0.19
Sulfonate	0.06 ± 0.05	0.03	0–0.15	0.07 ± 0.05	0.06	0–0.22
Pregnanetriol ^b^	0.69 ± 0.46	0.54	0.18–1.88	1.10 ± 0.48	1.08	0.09–2.26
Glucuronide ^c^	0.40 ± 0.44	0.23	0.02–1.88	0.74 ± 0.49	0.65	0.01–1.98
Sulfonate	0.29 ± 0.19	0.25	0–0.77	0.35 ± 0.27	0.29	0–1.02
d5-pregnanetriol	0.10 ± 0.17	0.05	0.01–0.80	0.10 ± 0.08	0.07	0.01–0.29
Glucuronide	0.10 ± 0.17	0.05	0.01–0.80	0.10 ± 0.08	0.07	0.01–0.29
Sulfonate	-	-	-	-	-	0–0.03
Pregnanetriolone	0.19 ± 0.16	0.17	0.01–0.55	0.22 ± 0.15	0.19	0.02–0.72
Glucuronide ^a^	0.08 ± 0.10	0.04	0.01–0.45	0.12 ± 0.13	0.09	0.01–0.72
Sulfonate	0.11 ± 0.11	0.10	0–0.38	0.10 ± 0.07	0.10	0–0.22

^a^ *p* < 0.05; ^b^ *p* < 0.01; ^c^ *p* ≤ 0.001.

**Table 5:** Significant correlations among urinary 17-KS, pregnanes and their respective glucuronidated compounds and total PCBs in smokers and non-smokers + ex-smokers, analyzed separately.

Hormones	Total PCBs	
	Smokers (N = 20)	Non-Smokers + Ex-Smokers (N = 36)
Total 17-KS	0.01	−0.45 ^b^
Glucuronides	0.06	−0.40 ^a^
Androsterone	−0.15	−0.52 ^c^
Glucuronide	−0.07	−0.40 ^a^
Etiocholanolone	−0.05	−0.36 ^a^
DHEA	0.11	−0.44 ^b^
Glucuronide	0.07	−0.34 ^a^
Total Pregnanes	0.12	−0.38 ^a^
Glucuronides	0.14	−0.33 ^a^
d5-pregnanediol	0.23	−0.35 ^a^
Glucuronide	0.13	−0.35 ^a^

17-ketosteroids; DHEA: dehydroepiandrosterone; ^a^ *p* < 0.05; ^b^ *p* ≤ 0.01; ^c^ *p* = 0.001.

**Table 6:** Influence of exposure to PCBs and cigarette smoking on total urinary 17-KS and 17-KS glucuronides assessed by two-way analysis of variance.

Hormones	Exposure to PCBs	Smokers				Non-Smokers + Ex-Smokers				
		N	Mean ± SD	Median	Range	N	Mean ± SD	Median	Range	ANOVA
Total 17-KS	Yes No	6 14	7.97 ± 5.74 10.59 ± 3.69	6.52 9.63	2.15–14.68 4.80–17.49	20 16	5.59 ± 2.35 9.23 ± 4.75	5.78 8.50	1.58–10.58 1.38–21.85	Model: *p* ≤ 0.01 Smoke: *p* ≤ 0.01 PCB: NS
Glucuronides	Yes No	6 14	5.71 ± 5.06 7.98 ± 3.50	3.47 6.93	1.24–13.81 3.43–15.48	20 16	3.35 ± 2.48 5.86 ± 3.92	2.97 4.67	0.25–9.17 0.16–17.68	Model: *p* ≤ 0.01 Smoke: *p* < 0.05 PCB: *p* < 0.05
Androsterone	Yes No	6 14	2.88 ± 2.12 3.99 ± 1.41	2.55 3.61	0.66–5.67 1.90–6.09	20 16	2.40 ± 1.01 3.37 ± 2.15	2.54 2.82	0.88–4.71 0.74–10.30	Model: *p* < 0.05 Smoke: *p* < 0.05 PCB: NS
Etiocholanolone	Yes No	6 14	2.17 ± 1.63 3.34 ± 1.43	1.69 3.22	0.63–4.40 1.11–6.67	20 16	1.46 ± 0.75 2.21 ± 0.82	1.48 2.34	0.41–3.09 0.24–3.32	Model: *p* < 0.001 Smoke: *p* ≤ 0.01 PCB: *p* ≤ 0.01
Glucuronide	Yes No	6 14	2.02 ± 1.60 2.93 ± 1.28	1.46 2.67	0.48–4.40 0.99–5.81	20 16	1.12 ± 0.79 1.82 ± 0.77	0.97 1.93	0.13–3.09 0.05–3.07	Model: *p* < 0.001 Smoke: *p* < 0.05 PCB: *p* ≤ 0.01
11-hydroxyandrosterone	Yes No	6 14	1.84 ± 1.23 2.07 ± 0.68	1.82 2.14	0.40–3.74 0.83–3.18	20 16	1.32 ± 0.56 2.58 ± 2.11	1.42 2.22	0.17–2.57 0.28–8.84	Model: *p* < 0.05 Smoke: NS PCB: NS
11-ketoetiocholanolone	Yes No	6 14	0.59 ± 0.51 0.82 ± 0.32	0.51 0.87	0.09–1.42 0.29–1.32	20 16	0.30 ± 0.22 0.82 ± 0.63	0.26 0.74	0.05–0.87 0.10–2.73	Model: *p* ≤ 0.01 Smoke: *p* ≤ 0.01 PCB: NS
Glucuronide	Yes No	6 14	0.46 ± 0.55 0.69 ± 0.28	0.17 0.59	0.06–1.40 0.29–1.32	20 16	0.22 ± 0.18 0.63 ± 0.64	0.18 0.47	0.01–0.71 0.02–2.73	Model: *p* ≤ 0.01 Smoke: *p* < 0.05 PCB: NS

17-KS: 17-ketosteroids. NS = Not significant.

**Table 7:** Influence of exposure to PCBs and cigarette smoke on total urinary pregnanes and glucuronides assessed by two-way analysis of variance.

Hormones	Exposure to PCBs	Smokers				Non-Smokers + Ex-Smokers				
		N	Mean ± SD	Median	Range	N	Mean ± SD	Median	Range	ANOVA
Total Pregnanes	Yes No	6 14	2.47 ± 2.05 2.84 ± 1.11	1.82 2.75	0.69–5.64 1.29–4.75	20 16	1.42 ± 0.84 2.36 ± 1.15	1.37 2.24	0.37–3.42 0.22–4.66	Model: *p* ≤ 0.01 Smoke: NS PCB: *p* < 0.05
Glucuronides	Yes No	6 14	1.89 ± 1.89 2.15 ± 1.05	1.06 1.76	0.50–5.26 0.85–4.33	20 16	0.85 ± 0.72 1.62 ± 1.03	0.67 1.56	0.09–2.49 0.09–4.35	Model: *p* ≤ 0.01 Smoke: NS PCB: *p* < 0.05
Pregnanediol	Yes No	6 14	0.49 ± 0.48 0.44 ± 0.25	0.32 0.32	0.12–1.34 0.188–0.88	20 16	0.18 ± 0.10 0.31 ± 0.18	0.20 0.30	0.05–0.37 0.04–0.70	Model: *p* ≤ 0.01 Smoke: NS PCB: *p* ≤ 0.01
Glucuronide	Yes No	6 14	0.45 ± 0.47 0.38 ± 0.23	0.28 0.29	0.10–1.28 0.14–0.78	20 16	0.13 ± 0.08 0.25 ± 0.16	0.12 0.20	0.02–0.28 0.02–0.62	Model: *p* ≤ 0.01 Smoke: NS PCB: *p* < 0.001
α-d5-pregnanediol	Yes No	6 14	0.41 ± 0.24 0.61 ± 0.46	0.37 0.51	0.06–0.74 0.13–1.90	20 16	0.23 ± 0.34 0.51 ± 0.34	0.11 0.46	0.01–1.56 0.04–1.24	Model: *p* < 0.05 Smoke: *p* < 0.05 PCB: NS
d5-pregnanediol	Yes No	6 14	0.15 ± 0.12 0.16 ± 0.07	0.13 0.15	0.03–0.29 0.07–0.32	20 16	0.08 ± 0.06 0.14 ± 0.09	0.07 0.14	0.01–0.18 0.01–0.33	Model: *p* < 0.05 Smoke: NS PCB: *p* < 0.05
Glucuronide	Yes No	6 14	0.07 ± 0.06 0.09 ± 0.04	0.05 0.08	0.01–0.15 0.01–0.19	20 16	0.04 ± 0.03 0.08 ± 0.05	0.02 0.07	<0.01–0.13 0.01–0.18	Model: *p* < 0.05 Smoke: *p* < 0.05 PCB: NS
Pregnanetriol	Yes No	6 14	0.85 ± 0.68 1.21 ± 0.50	0.69 1.23	0.18–1.88 0.37–2.26	20 16	0.64 ± 0.38 1.00 ± 0.46	0.55 0.92	0.20–0.52 0.09–1.65	Model: *p* ≤ 0.01 Smoke: *p* < 0.05 PCB: NS
Glucuronide	Yes No	6 14	0.59 ± 0.69 0.88 ± 0.53	0.27 0.74	0.14–1.88 0.22–1.98	20 16	0.34 ± 0.34 0.62 ± 0.43	0.23 0.55	0.02–1.43 0.01–1.65	Model: *p* < 0.05 Smoke: *p* < 0.05 PCB: NS

NS = not significant.

## Supplementary Materials

The following are available online at www.mdpi.com/1660-4601/13/4/360/s1, Table S1: Urinary concentrations of 17-KS in exposed subjects and controls subdivided by smoking habit, Table S2: Urinary concentrations of pregnanes in exposed subjects and controls subdivided by smoking habit.
