# Supplementary material for: Influence of Occupational and Environmental Exposure to Low Concentrations of Polychlorobiphenyls and a Smoking Habit on the Urinary Excretion of Corticosteroid Hormones
Source: Int J Environ Res Public Health. 2016 Mar 25;13(4):360. doi: 10.3390/ijerph13040360 (PMC4847022; doi:10.3390/ijerph13040360)
Supplement: Supplementary file 1 [file ijerph-13-00360-s001.pdf]

# Supplementary Materials: Influence of Occupational and Environmental Exposure to Low Concentrations of Polychlorobiphenyls and a Smoking Habit on the Urinary Excretion of Corticosteroid Hormones

**Maria Nicolà D'Errico, Piero Lovreglio, Ignazio Drago, Pietro Apostoli and Leonardo Soleo**

**Table S1.** Urinary concentrations of 17-KS in exposed subjects and controls subdivided by smoking habit.

| 17-KS (mg/L) | Smokers ($N$ = 20) | | | Non Smokers + Ex Smokers ($N$ = 36) | | |
|---|---|---|---|---|---|---|
| | Mean ± SD | Median | Range | Mean ± SD | Median | Range |
| Total [a] | 9.80 ± 4.42 | 9.27 | 2.15–17.49 | 7.21 ± 4.01 | 6.46 | 1.38–21.85 |
| Glucuronides [b] | 7.30 ± 4.03 | 6.48 | 1.24–15.48 | 4.47 ± 3.39 | 4.03 | 0.16–17.68 |
| Sulfonates | 2.50 ± 2.12 | 1.65 | 0.18–9.21 | 2.74 ± 1.60 | 2.65 | 0.15–7.36 |
| Androsterone [a] | 3.66 ± 1.68 | 3.58 | 0.66–6.09 | 2.83 ± 1.66 | 2.68 | 0.74–10.30 |
| Glucuronide [a] | 2.79 ± 1.65 | 2.48 | 0.37–6.09 | 1.77 ± 1.47 | 1.44 | 0.06–7.71 |
| Sulfonate | 0.87 ± 0.92 | 0.52 | 0–3.92 | 1.06 ± 0.58 | 0.99 | 0–2.59 |
| Etiocholanolone [b] | 2.99 ± 1.55 | 2.68 | 0.63–6.67 | 1.79 ± 0.85 | 1.78 | 0.24–3.32 |
| Glucuronide [c] | 2.66 ± 1.41 | 2.32 | 0.48–5.81 | 1.43 ± 0.85 | 1.52 | 0.05–3.09 |
| Sulfonate | 0.33 ± 0.48 | 0.18 | 0–2.04 | 0.36 ± 0.30 | 0.27 | 0.0–1.23 |
| DHEA | 0.31 ± 0.46 | 0.09 | 0.01–1.63 | 0.16 ± 0.31 | 0.06 | 0.01–1.74 |
| Glucuroniede [a] | 0.11 ± 0.12 | 0.06 | 0.01–0.43 | 0.05 ± 0.07 | 0.03 | <0.01–0.32 |
| Sulfonate | 0.20 ± 0.35 | 0.04 | 0–1.25 | 0.11 ± 0.26 | 0.03 | 0–1.42 |
| 11-hydroxyandrosterone | 2.00 ± 0.85 | 1.98 | 0.40–3.74 | 1.86 ± 1.55 | 1.47 | 0.17–8.84 |
| Glucuronide [a] | 1.06 ± 0.80 | 0.82 | 0.06–3.02 | 0.79 ± 1.00 | 0.56 | 0.01–4.47 |
| Sulfonate | 0.94 ± 0.59 | 0.83 | 0.02–2.32 | 1.07 ± 0.92 | 0.85 | 0–5.00 |
| 11-ketoandrosterone [b] | 0.11 ± 0.07 | 0.09 | 0.03–0.26 | 0.09 ± 0.15 | 0.05 | 0.01–0.76 |
| Glucuronide [a] | 0.07 ± 0.6 | 0.04 | 0.01–0.20 | 0.04 ± 0.06 | 0.02 | <0.01–0.36 |
| Sulfonate | 0.04 ± 0.04 | 0.03 | 0–0.14 | 0.05 ± 0.13 | 0.01 | 0–0.75 |
| 11-ketoetiocholanolone [a] | 0.75 ± 0.39 | 0.72 | 0.09–1.42 | 0.53 ± 0.52 | 0.38 | 0.05–2.73 |
| Glucuronide [b] | 0.62 ± 0.38 | 0.56 | 0.06–1.40 | 0.40 ± 0.48 | 0.24 | 0.01–2.73 |
| Sulfonate | 0.13 ± 0.20 | 0.01 | 0–0.74 | 0.13 ± 0.21 | 0.08 | 0–1.13 |

17-KS: 17-ketosteroids; DHEA: dehydroepiandrosterone; [a] $p \leq 0.05$; [b] $p \leq 0.01$; [c] $p \leq 0.001$.

**Table S2.** Urinary concentrations of pregnanes in exposed subjects and controls subdivided by smoking habit.

| PREGNANES (mg/L) | Smokers ($N$ = 20) | | | Non Smokers + Ex Smokers ($N$ = 36) | | |
|---|---|---|---|---|---|---|
| | Mean ± SD | Median | Range | Mean ± SD | Median | Range |
| Total [b] | 2.73 ± 1.41 | 2.56 | 0.69–5.64 | 1.84 ± 1.08 | 1.66 | 0.22–4.66 |
| Glucuronides [b] | 2.07 ± 1.30 | 1.68 | 0.50–5.26 | 1.19 ± 0.94 | 1.05 | 0.09–4.35 |
| Sulfonates | 0.66 ± 0.46 | 0.56 | 0.02–1.68 | 0.64 ± 0.39 | 0.55 | 0.12–2.19 |
| Pregnanediol [b] | 0.46 ± 0.33 | 0.32 | 0.12–0.34 | 0.24 ± 0.15 | 0.21 | 0.04–0.70 |
| Glucuronide [b] | 0.40 ± 0.31 | 0.28 | 0.10–1.28 | 0.18 ± 0.13 | 0.16 | 0.02–0.62 |
| Sulfonate | 0.06 ± 0.05 | 0.04 | 0–0.19 | 0.06 ± 0.05 | 0.04 | 0–0.20 |
| $\alpha$-pregnanediol | 0.14 ± 0.14 | 0.11 | 0.01–0.60 | 0.10 ± 0.11 | 0.06 | 0.01–0.61 |
| Glucuronide | 0.09 ± 0.07 | 0.06 | 0.01–0.21 | 0.07 ± 0.06 | 0.05 | 0.01–0.26 |
| Sulfonate | 0.05 ± 0.10 | 0.02 | 0–0.39 | 0.03 ± 0.06 | 0.01 | 0–0.35 |
| $\alpha$-d5-pregnanediol [a] | 0.55 ± 0.41 | 0.49 | 0.06–1.90 | 0.35 ± 0.36 | 0.19 | 0.01–1.56 |
| Glucuronide [b] | 0.47 ± 0.39 | 0.39 | 0.04–1.90 | 0.27 ± 0.31 | 0.15 | 0.01–1.29 |
| Sulfonate | 0.08 ± 0.09 | 0.06 | 0–0.39 | 0.08 ± 0.14 | 0.04 | 0–0.77 |
| d5-pregnanediol [a] | 0.16 ± 0.09 | 0.15 | 0.03–0.32 | 0.11 ± 0.08 | 0.11 | 0.01–0.33 |
| Glucuronide [a] | 0.08 ± 0.05 | 0.08 | 0.01–0.19 | 0.06 ± 0.05 | 0.04 | <0.01–0.18 |
| Sulfonate | 0.08 ± 0.05 | 0.08 | 0.0–0.15 | 0.05 ± 0.05 | 0.03 | 0–0.22 |
| Pregnanetriol | 1.10 ± 0.57 | 1.10 | 0.18–2.26 | 0.80 ± 0.45 | 0.70 | 0.09–1.65 |
| Glucuronide [a] | 0.80 ± 0.58 | 0.70 | 0.14–1.98 | 0.46 ± 0.41 | 0.38 | 0.01–1.65 |
| Sulfonate | 0.30 ± 0.27 | 0.20 | 0–0.90 | 0.33 ± 0.22 | 0.29 | 0–1.02 |
| d5-pregnanetriol | 0.14 ± 0.17 | 0.12 | 0.01–0.80 | 0.08 ± 0.09 | 0.05 | 0.01–0.32 |
| Glucuronide | 0.14 ± 0.17 | 0.12 | 0.01–0.80 | 0.08 ± 0.09 | 0.05 | 0.01–0.32 |
| Sulfonate | - | - | - | - | - | - |
| Pregnanetriolone | 0.22 ± 0.15 | 0.19 | 0.01–0.55 | 0.20 ± 0.16 | 0.16 | 0.01–0.72 |
| Glucuronide | 0.12 ± 0.09 | 0.10 | 0.01–0.45 | 0.10 ± 0.13 | 0.05 | 0.01–0.72 |
| Sulfonate | 0.11 ± 0.10 | 0.10 | 0–0.36 | 0.10 ± 0.09 | 0.10 | 0–0.38 |

[a] $p < 0.05$; [b] $p \leq 0.01$.

© 2016 by the authors; licensee MDPI, Basel, Switzerland. This article is an open access article distributed under the terms and conditions of the Creative Commons by Attribution (CC-BY) license (http://creativecommons.org/licenses/by/4.0/).
